# Sequential Combined Effect of Phages and Antibiotics on the Inactivation of *Escherichia coli*

**DOI:** 10.3390/microorganisms6040125

**Published:** 2018-12-05

**Authors:** Ana Lopes, Carla Pereira, Adelaide Almeida

**Affiliations:** Department of Biology and CESAM, University of Aveiro, Campus Universitário de Santiago, 3810-193 Aveiro, Portugal; lopes.sofia@ua.pt (A.L.); csgp@ua.pt (C.P.)

**Keywords:** Phage therapy, ciprofloxacin, combination therapies, *Escherichia coli*, resistance

## Abstract

The emergence of antibiotic resistance in bacteria is a global concern. The use of bacteriophages (or phages) alone or combined with antibiotics is consolidating itself as an alternative approach to inactivate antibiotic-resistant bacteria. However, phage-resistant mutants have been considered as a major threat when phage treatment is employed. *Escherichia coli* is one of the main responsible pathogens for moderate and serious infections in hospital and community environments, being involved in the rapid evolution of fluoroquinolones and third-generation cephalosporin resistance. The aim of this study was to evaluate the effect of combined treatments of phages and antibiotics in the inactivation of *E. coli*. For this, ciprofloxacin at lethal and sublethal concentrations was added at different times (0, 6, 12 and 18 h) and was tested in combination with the phage ELY-1 to inactivate *E. coli*. The efficacy of the combined treatment varied with the antibiotic concentration and with the time of antibiotic addition. The combined treatment prevented bacterial regrowth when the antibiotic was used at minimum inhibitory concentration (MIC) and added after 6 h of phage addition, causing less bacterial resistance than phage and antibiotic applied alone (4.0 × 10^−7^ for the combined treatment, 3.9 × 10^−6^ and 3.4 × 10^−5^ for the antibiotics and the phages alone, respectively). Combined treatment with phage and antibiotic can be effective in reducing the bacterial density and it can also prevent the emergence of resistant variants. However, the antibiotic concentration and the time of antibiotic application are essential factors that need to be considered in the combined treatment.

## 1. Introduction

*Escherichia coli* is a non-pathogenic commensal bacterium characterized by its diversity and versatility since it is able to colonize human and other animal gastrointestinal systems. However, this species developed some variants that colonize outside the gastrointestinal system. These strains harbor many virulence factors, causing severe diseases such as diarrhea, urinal tract infections, septicemia, pneumonia and meningitis [1]. *E. coli* strains involved in enteric disease are divided into enterohaemorrhagic *E. coli* (EHEC), enteropathogenic *E. coli* (EPEC), enterotoxigenic *E. coli* (ETEC), Shiga-toxin-producing enteroaggregative *E. coli* (STEAEC), enteroaggregative *E. coli* (EAEC), enteroinvasive *E. coli* (EIEC), adherent invasive *E. coli* (AIEC) and diffusely adhering *E. coli* (DAEC) [2,3,4,5]. The extraintestinal pathogenic *E. coli* is divided into neonatal meningitis-associated *E. coli*, uropathogenic *E. coli* (UPEC) and sepsis-causing *E. coli* [6]. Additionally, the development of antibiotics resistance within intestinal and extra-intestinal *E. coli* strains is currently increasing, especially against beta-lactam antibiotics and quinolones [7,8]. According to the World Health Organization (WHO), one of the most problematic areas of drug resistance is the rapid evolution of fluoroquinolones and third-generation cephalosporin resistance in Enterobacteriaceae. This is problematic in *E. coli*, which is the main representative species of this family [9,10]. Actually, *E. coli* strains, specifically antibiotic resistant ones, are among the main bacteria responsible for moderate and serious infections in the hospital and community environments [1,7].

Infections with resistant bacteria, namely those caused by Gram-negative bacteria, are difficult to treat, causing severe illness and requiring costly and sometimes toxic alternatives such as last resort antibiotics such as colistin, which is used against Gram-negative bacteria. Nevertheless, bacterial strains resistant to last resort antibiotics have been isolated worldwide [11,12]. The development of new antibiotics is not likely to solve the problem and it is probably only a matter of time until they become ineffective. Bacteria will inevitably find ways of resisting the conventional antibiotics, which is why new approaches are urgent to be used as an alternative to antibiotics.

The use of phages as antibacterial agents can be a very promising alternative for the treatment of infections, to be used alone or in combination with antibiotics. Phage treatment is based on the use of lytic phages to combat bacterial infections, mainly those caused by multidrug-resistant bacteria. There are several potential advantages of the application of phage therapy over antibiotics. Phages are usually highly specific to a single species or even strain of bacteria and therefore cause much less damage to the normal flora. Phages have limited impact in natural bacterial communities because they are self-replicating as well as self-limiting, they replicate exponentially as bacteria and decline when bacteria number decreases [13]. Until the advent of antibiotics, phage therapy was widely used, especially in Eastern Europe countries and, recently, the emergence of pathogenic bacteria resistant to antibiotics, including multidrug resistant bacteria, has motivated the western scientific community to reevaluate phage therapy as a valid option for the treatment of bacterial infections [14,15,16,17]. Currently, the potential use of phage therapy in agriculture, veterinary biocontrol, food safety and in clinical treatment of human infections is also being studied [18,19,20,21,22,23,24,25,26]. A few studies have demonstrated that phages can be used to successfully prevent or control *E. coli*, even antibiotic resistant strains [27,28,29] and, to the best of our knowledge, only one study (from our group) have been done using the combination of phages and antibiotics to prevent the emergence of phage-resistant *E. coli* mutants [30]. 

The emergence of phage-resistant mutants is nowadays a major concern regarding the use of phages to control bacterial infections. It has been shown that the use of phage cocktails can reduce the development of phage-resistant mutants [31,32,33,34] as well as the combined use of phages and antibiotics. Some studies report a synergetic effect of the combined use of antibiotics and phages [30,35,36,37,38,39,40,41,42,43,44,45,46,47,48], but only six of them show a reduced emergence of phage-resistant mutants by the combined effect of antibiotics and phages [30,40,46,49,50,51]. Oechslin et al. (2017) showed that the phages cocktail PP 1131 was active against *Pseudomonas aeruginosa* infection in endocarditis and highly synergistic with ciprofloxacin [40]. In this study, phage-resistant mutants regrew after 24 h but were prevented by the combination with ciprofloxacin. Escobar-Páramo et al. (2012) also observed a reduction in the emergence of phage-resistant *Pseudomonas fluorescens* SBW25 by the combined therapy [50]. According to Burrowes et al. (2011), the decrease in bacterial resistance to phages and/or antibiotics in dual therapy is due to the fact that a strain that is non-susceptible to one antimicrobial agent can be eliminated by the second one [51]. Torres-Barceló et al. (2016) showed a strong synergism effect of the combination of antibiotics and phages on *P. aeruginosa* PAO1 population density and in limiting its recovery rate [49]. In another study, Kirby (2012) observed that, after the treatment with a combination of gentamicin and phage SA5, *Staphylococcus aureus* phage-resistant isolates were extinct [46]. In a previous study, our group demonstrated that the efficacy of the combination of both therapies depends on the antibiotic resistance status of the targeted bacteria to the employed antibiotic and on the antibiotic type (bactericide or bacteriostatic), causing the same or less bacterial resistance than phages and antibiotics applied alone [30]. However, there are very few studies about the interaction process of the two therapies, namely, the effect of using different antibiotic/phage doses and antibiotic addition times on the development of resistance. The antibiotic dose can have an important effect on the development of resistance, but the mechanisms involved differ between low and high doses. Usually, lower doses are selected to avoid the development of resistance [52,53]. The application time is another factor influencing the short- and long-term efficiency of combined therapies, particularly for antibiotics and phage, where the phage replication and the effect of antibiotic are density-dependent [54]. The dynamic of phage population is determined by the number of available hosts, with consequences for the amplification of phage densities and for the therapeutic effectiveness [55]. If phages are administered to low bacterial densities or to bacteria non-amenable physiologically, the phage density will decrease leading to the possible need of a recurrent application of phages [55]. Thus, in this work, we studied the combined effect of an antibiotic and a phage on *E. coli* inactivation efficacy and on the development of resistance, testing different antibiotic application times and phage/antibiotic doses.

## 2. Materials and Methods

### 2.1. Bacterial Strains and Growth Conditions

The genetically transformed bacterium *E. coli* Top10 [56] was used as a bioluminescent bacterial model. This method allows monitoring in real-time the effect of phage therapy on *E. coli* through the measurement of bioluminescence, thus avoiding the laborious and time-consuming conventional method of counting colony-forming units (CFU).

Other bacterial strains were used to determine the phage host range: *Salmonella enterica* serovar Typhimurium (ATCC 13311 and ATCC 14028), *E. coli* (ATCC 25922 and ATCC 13706), *Aeromonas hydrophila* (ATCC 7966), *Vibrio fischeri* (ATCC 49387), *Vibrio parahaemolyticus* (DSM 27657), *Vibrio anguillarum* (DSM 21597), *Photobacterium damselae damselae* (DSM 7482), *Shigella flexneri* (DSM 4782), *Listeria innocua* (NCTC 11288), *Listeria monocytogenes* (NCTC 1194), *Aeromonas salmonicida* (CECT 894) and five strains of *Salmonella enterica* serovar Enteriditis. *Salmonella* Enteriditis was isolated from food (kindly provided by Controlvet Laboratory). *E. coli* (AE11, AN19, AD6, AF15, BC30, AC5, AJ23, BN65, and BM62), *Enterobacter cloacae*, *Citrobacter freundii*, *Proteus mirabilis*, *Providencia* sp. and *P. aeruginosa* were isolated in previous works from water samples collected in Ria de Aveiro [31,57]. The bioluminescent *E. coli*, used as phage host in the phage treatment experiments, was stored at −80 °C in 10% glycerol. Before each assay, a stock culture of *E. coli* was aseptically inoculated in 30 mL of Tryptic Soy Broth (TSB, Liofilchem, Italy) and was grown overnight at 25 °C and 120 rpm stirring. Then, an aliquot (300 µL) of this culture was transferred to 30 mL of fresh TSB and grown overnight at 25 °C under stirring (120 rpm), to reach 10^8^ relative light units (RLU), corresponding approximately to 8 log colony forming units (CFU)/mL.

The other bacterial strains used in this study were maintained in solid Tryptic Soy Agar (TSA; Liofilchem, Italy) at 4 °C. Before each assay, one isolated colony was aseptically transferred to 30 mL of TSB and incubated as described above. Then, an aliquot of each culture was subcultured in 30 mL of fresh TSB to reach an optical density (O.D.) of 600 nm = 0.8, corresponding to about 10^8–9^ cells per mL.

### 2.2. Correlation between Bioluminescence and CFU

An overnight culture of the bioluminescent *E. coli* (10^8^ CFU/mL) was serially diluted (10^−1^–10^−8^) in phosphate buffered saline (PBS, 137 mM NaCl (Sigma), 2.7 mM KCl (Sigma), 8.1 mM Na_2_HPO_4_·2H_2_O, and 1.76 mM KH_2_PO_4_ (Sigma), pH 7.4). The non-diluted (10^0^) and diluted aliquots were read on a luminometer (1 mL) (TD-20/20 Luminometer, Turner Designs, Inc., Madison, WI, USA) to determine the bioluminescence signal. Simultaneously, 1 mL of each dilution was pour-plated in TSA and incubated at 25 °C for 24 h. Three independent experiments were performed and the results were averaged. 

### 2.3. Phage Preparation

The phage ELY-1 was isolated in a previous work from water samples collected from the Corte das Freiras Aquaculture [58]. The phage ELY-1 was identified as a T4-like phage with 95% of homology with the Enterobacteriaceae phage vB_EcoMVR7 (accession number HM563683) [58]. Silva et al. (2014) demonstrated that the phage ELY-1 was effective in reducing the bioluminescent *E. coli* strain [59].

The phage suspension was prepared from the phage stock previously prepared in SM buffer (0.1 M NaCl (Sigma), 8 mM MgSO_4_ (Sigma), 20 mM Tris-HCl (Sigma), 2% (*w*/*v*) gelatin, pH 7.5). Three hundred microliters of the phage stock were added to thirty milliliters of TSB with double concentration and one milliliter of the *E. coli* in exponential growth phase. The suspension was grown overnight and incubated at 25 °C at 50 rpm. The lysate was centrifuged at 13,000 rpm for 30 min at 4 °C to remove intact bacteria or bacterial debris. Phage suspension was stored at 4 °C and the titer was determined by the double-layer agar method [58]. Successive dilutions of the phage suspension were performed in PBS according to Pereira et al. (2011) [60] and plates were incubated at 25 °C for 12 h. After incubation, the number of plaques was counted and the results expressed as plaque-forming units (PFU)/mL.

### 2.4. Phage Host Range Determination and Efficiency of Plating (EOP) Analysis

Phage host range was determined by spot test according to Vieira et al. (2012) [61]. The plates were incubated at 25 °C and examined for plaques after 12 h. Bacterial sensitivity to the phage was established by a lysis cleared zone at the spot. According to the clarity of the spot, bacteria were differentiated into two categories: clear lysis zone (+) and not lysis zone (−). Then, efficiency of plating (EOP) was determined for bacteria with positive spot tests (occurrence of a clear lysis zone) using the double-layer agar method [58]. The EOP was calculated (average PFU on target bacteria/average PFU on host bacteria) according to Kutter [62]. For each phage, three independent experiments were performed.

### 2.5. Antibiotic Preparation

The bioluminescent *E. coli* is resistant to ampicillin, chloramphenicol, kanamycin, piperacillin and susceptible to ciprofloxacin. As ciprofloxacin showed a synergistic effect in our previous study [30], contrarily to the other tested antibiotics, this antibiotic was chosen to perform the combined experiments with the phage. 

A stock solution of ciprofloxacin (Cip, Sigma-Aldrich, St. Louis, MO, USA) was prepared following the manufacture instructions and stored in the freezer at −80 °C.

### 2.6. Determination of Minimum Inhibitory Concentration (MIC)

The minimum inhibitory concentration (MIC) for ciprofloxacin was determined in triplicate by the microdilution method in Mueller Hinton broth according to European Committee on Antimicrobial (EUCAST) [63]. Different concentrations of the antibiotic (0.10–0.50 μg/mL) were prepared by serial dilution in Mueller Hinton broth. The tubes were inoculated with 100 μL of the bioluminescent *E. coli*. A control of bacteria without antibiotic was also included. The MIC was defined as the lowest concentration that showed no growth in the Mueller Hinton broth. The results were interpreted according to EUCAST [64]. Three independent experiments were performed.

### 2.7. Kill Curves with Phage and Ciprofloxacin in Tryptic Soy Broth (TSB)

Bioluminescent *E. coli* inactivation was determined using the phage and ciprofloxacin at 1 × MIC, 2 × MIC, 1/5MIC and 1/10MIC, at a multiplicity of infection (MOI) of 1 and 100 in TSB. To obtain a MOI of 1, 6.5 µL of 10^8^ URLs of the overnight *E. coli* culture (final concentration 10^5^ URLs) and 12 µL of 10^8^ PFU/mL of the phage suspension (final concentration 10^5^) were inoculated in 30 mL of TSB (B + P). To obtain a MOI of 100, a fresh culture of bacteria was inoculated as described above and 300 µL of 10^8^ PFU/mL of the phage suspension (final concentration 10^7^ PFU/mL) were inoculated in sterilized glass Erlenmeyer flasks with 30 mL of TSB. When the assays were performed with ciprofloxacin (B + P + Cip), the same phage and bacterium concentrations were used. For these assays, three controls were included: bacterial control (BC), antibiotic control (B + CipC) and the phage control (PC). The bacterial control was inoculated with bioluminescent *E. coli* but not with phage, the phage control was inoculated with phage and without bacteria, the antibiotic control was inoculated with the bioluminescent *E. coli* and antibiotic. The controls and test samples were incubated at 25 °C and 50 rpm stirring. Aliquots of test sample and bacterial and phage controls were collected at time zero and after 6, 12, 18, 24 and 36 h of incubation for bioluminescence signal measurement (BC, B + P, B + CipC, and B + P + Cip) and phage quantification (PC, B + P, and B + P + Cip). The bioluminescence signal was measured in the luminometer (TD-20/20 Luminometer, Turner Designs, Inc., Madison, WI, USA) in triplicate. The phage titer was determined, in duplicate, through the double agar layer method and plates were incubated for 12 h at 25 °C and expressed in PFU/mL. Three independent experiments were performed.

### 2.8. Kill Curves with Different Ciprofloxacin Addition Times

Bioluminescent *E. coli* inactivation was determined using phage and ciprofloxacin at 1 × MIC and at MOI of 100 in TSB. The bacterial and phage suspension was inoculated as described (see Section 2.7), with a final concentration of 10^5^ CFU/mL and 10^7^ PFU/mL, respectively. When the assays were performed with ciprofloxacin (B + P + Cip), the concentrations of phage and bacterium described above were used (see Section 2.7). The ciprofloxacin was added at time zero and 6, 12 or 18 h after incubation. Three control samples were included: bacterial control (BC), phage control (PC) and antibiotic control (B + CipC). The controls and test samples were prepared as described above (see Section 2.7). All controls were incubated exactly in the same conditions as the test samples. Aliquots of test samples, bacterial and phage control were collected at time zero and after 6, 12, 18, 24 and 36 h of incubation. The phage titer and bioluminescence signal were determined as described above (see Section 2.7). Three independent experiments were performed. 

### 2.9. Determination of the Rate of Emergence of Bacterial Mutants

**Figure 1:**
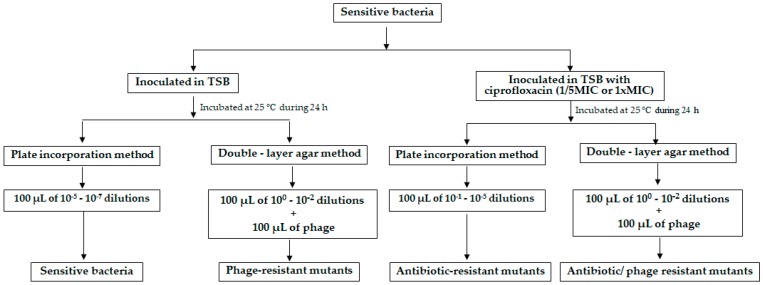
Design of the experimental work to test the emergence of bacterial resistances.

The development of resistant mutants of *E. coli* to phages, to ciprofloxacin at 1/5 MIC and 1 × MIC, to phage and ciprofloxacin at 1/5 MIC and 1 × MIC was evaluated according to Haddix et al. (2000) and Filippov et al. (2011) [65,66] (Figure 1). 

To determinate the frequency of phage-resistant bacteria, ten isolated colonies from a plate with sensitive bacteria were selected and inoculated into ten tubes with 5 mL of TSB, grown at 25 °C for 18 h. The ten previously prepared TSB cultures of bacteria were also used to determine the development of phage-resistant bacteria in the presence of ciprofloxacin. To determine the phage-resistant mutants, aliquots of 100 µL from the 10^0^ to 10^−2^ dilutions of the bacterial culture aliquots of 100 µL of the phage from a stock solution of 10^9^ PFU/mL were inoculated in tubes with TSB 0.6%, plated on TSA plates and incubated at 25 °C for 48 h. Simultaneously, 100 µL aliquots of 10^−5^ to 10^−7^ dilutions of the bacterial culture were plated by incorporation on TSA plates without phage or without phage and antibiotic and incubated at 25 °C for 24 h. The ten previously prepared TSB cultures of bacteria were also used to determine the phage-resistant mutants in the presence of ciprofloxacin. The same procedure was used, but the antibiotic was added to the cultures. To determinate the frequency of *E. coli* mutants resistant to the antibiotic (without phage) aliquots of 100 µL from the 10^−1^ to 10^−5^ dilutions of the bacterial culture were plated on TSA plates and incubated at 25 °C for 48 h. To determinate the frequency of *E. coli* mutants resistant to the antibiotic (with phage), aliquots of 100 µL from the 10^0^ to 10^−2^ dilutions of the bacterial culture were plated on TSA plates by double agar layer method and incubated at 25 °C for 48 h. The calculation of the frequency of mutants was done by dividing the number of resistant bacteria (obtained from the ten isolated colonies) by the total number of sensitive bacteria. This formula was used to calculate the frequency of antibiotic-resistant mutants, phage-resistant mutants and the frequency of mutants for the mixture of phage and antibiotic.

### 2.10. Statistical Analysis

Statistical analysis was performed using GraphPad Prism 7.04. Normal distribution was achieved by Kolmogorov–Smirnov test and homogeneity of variance was assessed by Levene’s test. A value of *p* < 0.05 was considered statistically significant and Tukey’s multiple comparison test was used for a pairwise comparison of the means. The existence of significant differences on bacterial concentration in killing curves assays was analyzed using a two-way analysis of variance (ANOVA) with repeated measures. The significance of the differences was evaluated by comparing the results obtained in the test samples and control samples for the different times between treatments of each of the three independent assays, along the incubation time. One-way ANOVA was used to examine differences between the concentration of resistant bacteria in the presence of the antibiotics, in the presence of the phage alone and in the presence of both simultaneously.

## 3. Results

### 3.1. Determination of Minimum Inhibitory Concentration (MIC)

The assessed MIC of the bioluminescent *E. coli* to ciprofloxacin was 0.25 mg/mL (MIC breakpoints: S ≤ 0.25 and R > 0.5 mg/mL, according to EUCAST [64]).

### 3.2. Phage Host Range and Efficiency of Plating (EOP) Analysis

Spot tests indicated that phage ELY-1 had the capacity to form completely cleared zones on five (*E. coli* bioluminescent, *E. coli* BC30, *E. coli* ATCC 25922, *S.* Typhimurium ATCC 13311 and *S.* Enteriditis CVD) of the 32 strains (Table 1). However, EOP results indicated that the phage formed phage lysis plaques on only two strains (*E. coli* ATCC 25922 and *S.* Typhimurium ATCC 13311) of the 32 strains tested. Phage infected *E. coli* ATCC 25922 and *S.* Typhimurium ATCC 13311 with an efficacy of 2.27 × 10^3^ and 3.45 × 10^−3^, respectively (Table 1).

### 3.3. Correlation between Bioluminescence and CFU

**Figure 2:**
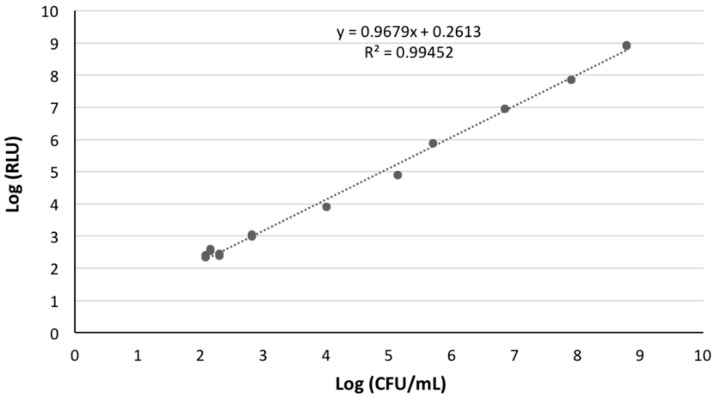
Association between the bioluminescence signal and viable counts of an overnight culture of a transformed bioluminescent *E. coli*. Bioluminescence is expressed in RLUs and viable counts in CFU/mL.

A linear correlation between viable counts and the bioluminescence signal of overnight cultures of the bioluminescent *E. coli* was observed (Figure 2).

### 3.4. Kill Curves with Phage and Ciprofloxacin in TSB

**Figure 3:**
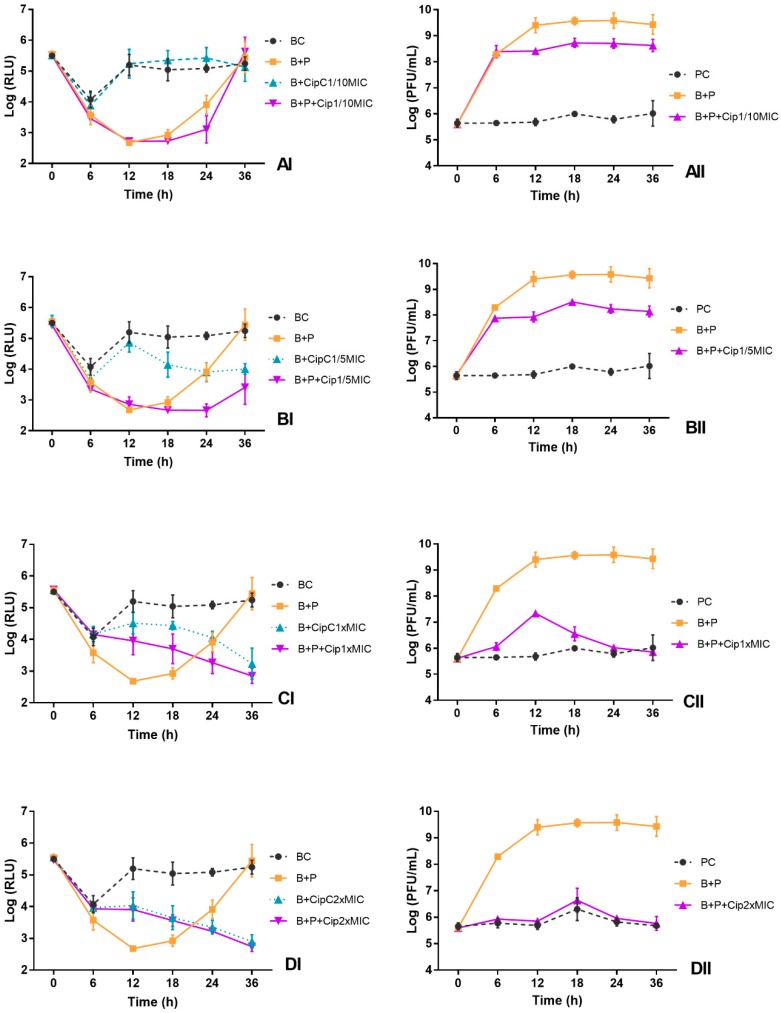
Effect of phage alone (MOI of 1) and of combined phage and ciprofloxacin treatments at different concentrations ((**A**) 1/10MIC, (**B**) 1/5 MIC, (**C**) MIC and (**D**) 2 × MIC) on the inactivation of bioluminescent *E. coli* (I) and the phage concentration (II) in TSB during 36 h. BC, bacterial control; PC, phage control; B + P, bacteria plus phage; B + CipC, bacteria plus ciprofloxacin; B + P + Cip, bacteria plus phage plus antibiotic. Values represent the mean of three independent experiments.

**Figure 4:**
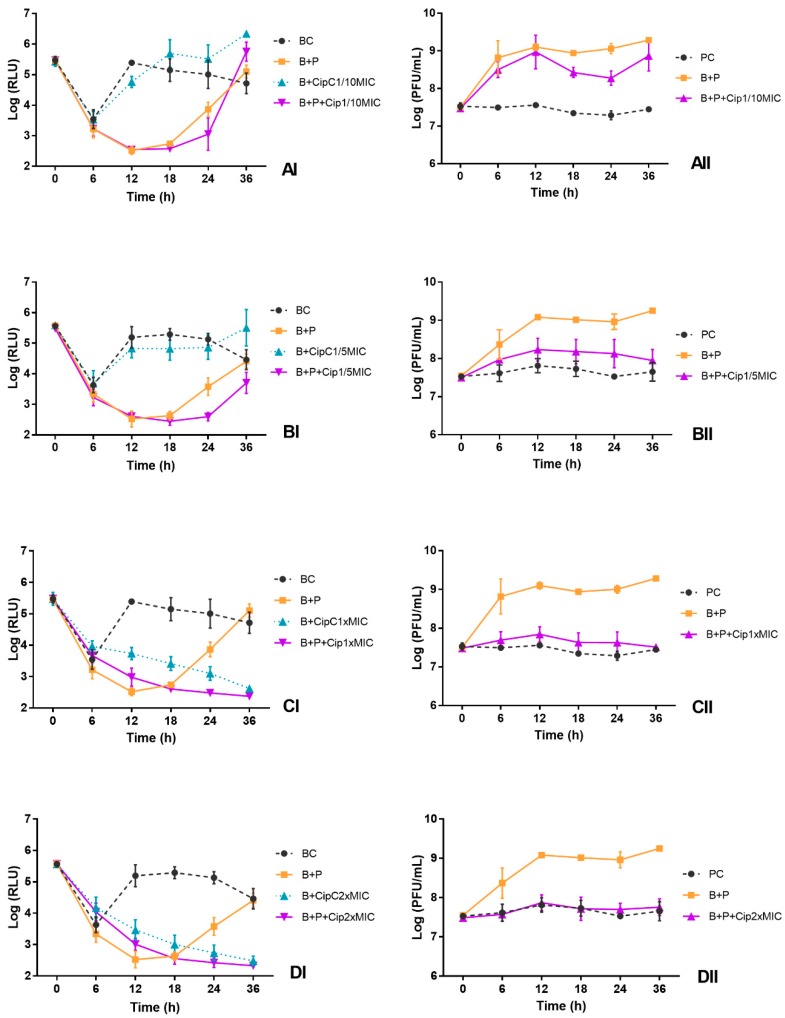
Effect of phage alone (MOI of 100) and of combined phage and ciprofloxacin treatments at different concentrations ((**A**) 1/10MIC, (**B**) 1/5MIC, (**C**) MIC and (**D**) 2 × MIC) on the inactivation of bioluminescent *E. coli* (I) and the phage concentration (II) in TSB during 36 h. BC, bacterial control; PC, Phage control; B + P, bacteria plus phage; B + CipC, bacteria plus ciprofloxacin; B + P + Cip, bacteria plus phage plus antibiotic. Values represent the mean of three independent experiments.

The bioluminescence signal of *E. coli* in the BC decreased 2 log RLU during the first 6 h of incubation (Figure 3AI–DI and Figure 4AI–DI). After 12 h of incubation, bacterial density remained constant until the end of treatment (Figure 3AI–DI and Figure 4AI–DI). The decrease of the bioluminescence signal during the first 6 h of incubation can be explained by the fact that the bacterium *E. coli* has been inoculated in TSB under non-controlled temperature. The bioluminescence signal of *E. coli* is temperature dependent, the light emission is linearly proportional to the numbers of colonies at temperatures below 25 °C.

The results of the experiments show that the phage ELY-1 was able to cause a decrease in the bioluminescence signal (ANOVA, *p* < 0.05) after 12 h of phage treatment (B + P), relative to the bacterial control (BC) (Figure 3AI–DI). The profile of variation was similar for both MOIs (by 2.5 log RLU for MOI of 1 and 2.8 log RLU after 12 h for MOI of 100). However, after this period, the bioluminescent *E. coli* regrowth was observed, reaching similar values (ANOVA, *p* > 0.05) to those obtained in the bacterial control after 36 h of incubation (Figure 3AI–DI).

When the phage ELY-1 was combined with antibiotic at sublethal concentrations (1/10 and 1/5 of MIC), the bioluminescent *E. coli* inactivation was similar in both MOIs (ANOVA, *p* > 0.05) (Figure 3AI,BI and Figure 4AI,BI). The maximum rate of bacterial inactivation when phage was combined with ciprofloxacin at 1/10 (B + P + Cip1/10MIC) and 1/5 of MIC (B + P + Cip1/5MIC), relative to the bacterial control, was 2.7 and 2.4 log RLU, respectively, achieved after 12 h of treatment (Figure 4AI,BI). However, after 36 h of incubation for both sublethal concentrations and for both MOIs, *E. coli* regrowth was observed, reaching bacterial densities similar (ANOVA, *p* > 0.05) to those obtained in bacterial control after 36 h of incubation (Figure 3AI,BI and Figure 4AI,BI). For this treatment, significant differences (ANOVA, *p* < 0.05) relative to the treatment with antibiotic alone at a sublethal concentration (B + CipC1/10MIC and B + CipC1/5MIC) were observed during 36 h of incubation. The bacterial inactivation in the treatment with antibiotic at a sublethal concentration (B + CipC1/10MIC and B + CipC1/5MIC) was significantly lower than that observed in the treatment with phage, with or without the antibiotic (ANOVA, *p* < 0.05). The bacterial inactivation in the treatment with phage and antibiotic at a sublethal concentration (B + P + Cip1/10MIC and B + P + Cip1/5MIC) was significantly higher (ANOVA, *p* < 0.05) than that observed when the phage was used alone (B + P) after 24 and 36 h of incubation. No decrease in bacterial inactivation was observed for the treatment with ciprofloxacin at these two sublethal concentrations (B + P + Cip1/5MIC and B + P + Cip1/10MIC) when compared with the bacterial control (BC). 

When the phage was combined with ciprofloxacin at lethal concentrations (B + P + Cip1 × MIC and B + P + Cip2 × MIC), the increase in the MOI from 1 to 100 promoted a decrease in *E. coli* bioluminescence after 12, 18 and 24 h of incubation (Figure 3CI,DI and Figure 4CI,DI, ANOVA, *p* < 0.05), but the pattern of variation between treatments was similar (ANOVA, *p* > 0.05) for both MOIs. 

The maximum rate of bacterial inactivation for the mix of the phage and ciprofloxacin at 1 × MIC (B + P + Cip1 × MIC) was 2.5 log RLU, achieved after 18 h of treatment at MOI of 100. In this treatment, as well as in the treatment with the antibiotic alone (B + CipC1 × MIC), no regrowth of bacteria was observed until the end of the treatment (Figure 3CI and Figure 4CI). When the phage was used alone, a significant regrowth, after 12 h of incubation, was observed (ANOVA, *p* < 0.05).

When the phage was combined with ciprofloxacin at 2 × MIC (B + P + Cip2 × MIC), the maximum rate of bacterial inactivation (2.7 log RLU, ANOVA, *p* < 0.05) was obtained after 18 h of incubation and was similar to that obtained in the antibiotic control (B + Cip2 × MIC) (Figure 3DI). During the treatment, no significant differences (ANOVA, *p* > 0.05) were observed for both MOIs between treatment with antibiotic alone (B + CipC2 × MIC) and treatment with antibiotic and phage (B + P + Cip2 × MIC) (Figure 3DI and Figure 4DI). However, the bacterial inactivation in the treatment with antibiotic, with (B + P + Cip2 × MIC) or without the phage (B + CipC2 × MIC), was significantly higher (ANOVA, *p* < 0.05) than that observed when the phage was used alone (B + P). When the phage was combined with antibiotic at 2 × MIC (B + P + Cip2 × MIC) or the antibiotic was used alone (B + CipC2 × MIC), no regrowth of bacteria was observed until the end of the treatment.

For both MOIs, the phage control (PC) remained constant throughout the experiment (ANOVA, *p* > 0.05), but, when the phage was incubated in the presence of the host without antibiotic, a significant increase (3.4 log PFU/mL for MOI of 1 and 1.8 log PFU/mL for MOI of 100, ANOVA, *p* < 0.05) was observed (Figure 3AII–DII and Figure 4AII–DII).

When the phage was incubated in the presence of the host (B + P) and antibiotic at sublethal concentrations (B + P + Cip1/10MIC and B + P + Cip1/5MIC), the phage concentration was significantly higher (ANOVA, *p* < 0.05) than that observed in the phage control (PC) (Figure 3AII,BII and Figure 4AII,BII). When the bacteria were treated with the phage in the presence of ciprofloxacin at sublethal concentrations, a significant increase (ANOVA, *p* < 0.05) was observed in the phage concentration (increase 2.0 log PFU/mL for MOI of 1 and 0.5–1.0 log PFU/mL for MOI of 100). However, for both MOIs, when the phage was incubated in the presence of the host and ciprofloxacin at lethal concentration (B + P + Cip1 × MIC and B + P + Cip2 × MIC), the phage concentration remained constant (ANOVA, *p* > 0.05) throughout the experiment (Figure 3CII,DII and Figure 4CII,DII). No significant differences (ANOVA, *p* > 0.05) between these treatments (B + P + Cip1 × MIC and B + P + Cip2 × MIC) and phage control (PC) were observed. 

### 3.5. Influence of Ciprofloxacin Addition Time on the Kill Curves

**Figure 5:**
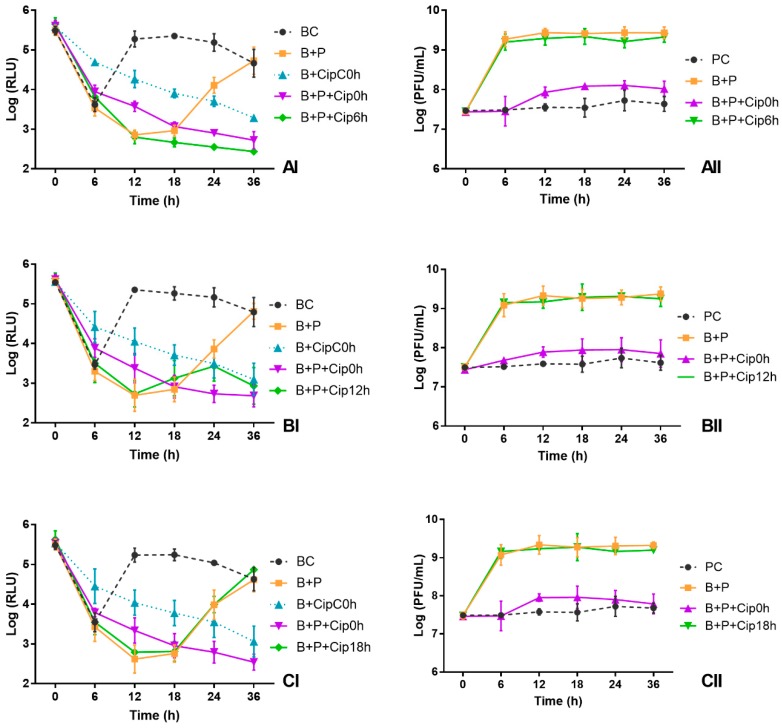
Effect of time of ciprofloxacin addition at 1 × MIC on the inactivation of bioluminescent *E. coli* (I) and the phage concentration (II) in TSB during 36 h: (**A**) antibiotic added after 6 h of phage addition; (**B**) antibiotic added after 12 h of phage addition; and (**C**) antibiotic added after 18 h of phage addition. BC, Bacterial control; PC, Phage control; B + P, Bacteria plus phage; B + CipC0h, Bacteria plus antibiotic added at the same time of the phage; B + P + Cip0h, Bacteria plus phage plus antibiotic added at the same time of the phage; B + P + Cip6h, Bacteria plus phage plus antibiotic added at 6 h of phage addition; B + P + Cip12h, Bacteria plus phage plus antibiotic added at 12 h of phage addition; B + P + Cip18h, Bacteria plus phage plus antibiotic added at 18 h of phage addition. Values represent the mean of three independent experiments.

After 6 h of incubation, the bioluminescence signal of *E. coli* in the BC decreased 2 log RLU, remaining constant until the end of treatment (Figure 5AI–DI). The bacterial addition to the TSB under non-controlled temperature affected the bioluminescence signal of *E. coli* during the first 6 h of incubation.

At the end of the treatment, the rate of bacterial inactivation when the antibiotic was added after 6 h of phage addition was 2.3 log RLU relative to the bacteria control, which was significantly higher (ANOVA, *p* < 0.05) than that obtained in the other tested conditions (B + P, B + P + Cip0h, B + P + Cip12h and B + P + Cip18h) (Figure 5AI–CI). When the antibiotic was added after 6 h of phage addition, no regrowth of bacteria was observed until the end of the treatment, contrarily to what was observed for the other two conditions (B + P and B + P + Cip18h).

When the antibiotic was added after 12 h of phage addition, the bacterial inactivation, after 36 h of incubation, decreased 1.9 log RLU relative to the bacteria control (BC) and was similar (ANOVA, *p* > 0.05) to that obtained when the antibiotic was added at the same time of the phage (decrease 2.1 log RLU). However, at the end of the treatment, the bacterial inactivation in this treatment was significantly higher (ANOVA, *p* < 0.05) than that obtained in treatment with phage without antibiotic (bacterial concentration was similar to that of the bacteria control) (Figure 5AI–CI).

When the antibiotic was added after 18 h of phage addition, after 36 h of treatment, the bacterial density was similar (ANOVA, *p* > 0.05) to that obtained in bacteria control (BC) and to that obtained in the treatment with phage without antibiotic (B + P) (Figure 5CI). In this condition, until 18 h of incubation, bacterial inactivation was similar to that obtained in B + P (ANOVA, *p* > 0.05) (Figure 5CI). 

The phage control (PC) remained constant throughout the experiment (ANOVA, *p* > 0.05) (Figure 5AII–CII). When the bacteria were incubated in presence of the phage without antibiotic (B + P), a significant increase (increase 1.8 log PFU/mL, ANOVA, *p* < 0.05) was observed in the phage concentration (Figure 5AII–CII). 

When the bacteria were treated with the phage in the presence of ciprofloxacin added 6, 12 and 18 h after phage addition (B + P + Cip6h B + P + Cip12h and B + P + Cip18h), a significant increase was observed in the phage concentration (increase 1.7–2.0 log PFU/mL, ANOVA, *p* < 0.05). The number of produced phages in the three conditions (B + P + Cip6h B + P + Cip12h and B + P + Cip18h) was similar (ANOVA, *p* > 0.05) to that obtained without the antibiotic addition (B + P) and significantly higher than that observed when the antibiotic was added at the same time of the phage (increase 0.5 log PFU/mL, relative to phage control) (Figure 5AII–CII). 

### 3.6. Determination of the Emergence Rate of Bacterial Mutants

The bioluminescent *E. coli* showed different rates of emergence of resistant mutants when subjected to ciprofloxacin alone, phage alone and to the mix of the phage plus ciprofloxacin (Table 2). The development of resistant mutants of *E. coli* against the ciprofloxacin alone at 1/5 MIC (5.24 × 10^−1^) was significantly higher (ANOVA, *p* < 0.05) than that obtained when phage was used alone (3.43 × 10^−5^) and when the mixture of phage and ciprofloxacin (4.00 × 10^−5^) was used. However, when bioluminescent *E. coli* was treated with ciprofloxacin at 1 × MIC (3.95 × 10^−6^), the rates of emergence of resistant mutants were significantly different (ANOVA, *p* < 0.05) from those obtained when phage was used alone (3.43 × 10^−5^) and when the mixture of phage and ciprofloxacin at 1 × MIC (4.04 × 10^−7^) was used. The frequency of *E. coli* resistant mutants in the presence of the phage alone (3.43 × 10^−5^) was similar (ANOVA, *p* > 0.05) to that obtained when the phage was used in combination with ciprofloxacin at 1/5MIC (4.00 × 10^−5^). However, the frequency of *E. coli* resistant mutants in the presence of the phage alone was significantly higher (ANOVA, *p* < 0.05) than that obtained when the phage was used in combination with ciprofloxacin at 1 × MIC (4.04 × 10^−7^).

## 4. Discussion

With the emergence of antibiotic resistance in common bacteria, such as *E. coli*, even in commensal strains, there is a need to develop alternative treatments. Several studies have demonstrated that phages can be used to control pathogenic bacteria [61,67,68,69,70,71,72,73,74] but the development of phage-resistant mutants is a general shortcoming [31,33,75,76]. The combination of phage treatment with antibiotics is a possibility to avoid the emergence of resistance, but little is known about the interaction process of phages and antibiotics in combined therapies, particularly in regard to the emergence of phage-resistant mutants. In a previous work of our group [30], we showed that the phage ECA2 and ciprofloxacin combination could result in synergistic effects against *E. coli*; nevertheless, bacterial regrowth after treatment was observed. Thus, in the present study, we try to understand if the development of bacterial regrowth can be circumvented by the use of different antibiotic and phage concentration combinations and also by the application of antibiotic after different times of phage addition. We showed that: (1) phage and antibiotic combinations could result in positive effects in the inactivation of bacteria, preventing bacterial regrowth; (2) the efficacy of the combination depends greatly on the concentration of antibiotic and on the time of antibiotic addition; (3) the efficacy of the combination is not greatly influenced by the initial phage concentration; and (4) the combination effectively decreases the development of bacterial resistance, not only against the antibiotic but also against the phage.

Phage specificity is one of the major advantages of phage treatment, since the non-target bacterial populations should remain undisturbed. The phage ELY-1 forms completely cleared zones on 5 of the 34 tested strains. However, EOP results indicate that the phage only forms lysis plaques on two of the tested bacterial strains. Other authors such as Mirzaei and Nilsson (2015) obtained similar results, stating that the spot test cannot be used for identification and selection of phages and should be replaced by the EOP assays [77]. Positive spot test and negative EOP results can happen when an overload of phages simultaneously infects a bacterium leading to lysis due to the presence of high concentrations of lysins [78]. In this situation, the bacteria can be inactivated before replicating the phages, which is known as “lysis from without”, due to a high concentration of phage lytic enzymes [79,80,81,82], not allowing to reach an enough number of phages to inactivate the non-enzyme lysed bacteria. Our results confirm that EOP should be used to evaluate the phage host range and show that the phage ELY-1 has a high specificity.

The kinetic theory indicates that the MOI could be critical to control pathogenic bacteria [83]. Several studies demonstrated that the reduction of pathogenic bacteria increases in parallel with MOI or that bacterial reduction occurs sooner at high MOI values [84,85,86]. However, Nakai (2010) demonstrated that the concentration of phage may not be crucial due to its self-perpetuating nature, responsible by an increasing of phage titers along with bacteria [79]. In this study, in general, the increase in MOI from 1 to 100 did not promote a significant increase in the efficiency of phage treatment. Nevertheless, when phage was combined with ciprofloxacin at lethal concentrations (1 × MIC and 2 × MIC), the increase of the MOI caused a decrease in bacterial density. This can be explained by the fact that, in the presence of the high antibiotic concentrations, the host DNA replication inhibition avoids the phage replication by the bacteria. If inhibition reduces the per-host cell output of phage, then the overall phage titer may not be sufficient to cause massive reductions in bacterial cell density when the phage is added at low MOI of 1. At MOI of 100, even if the host DNA replication is affected, the high number of initial phages present from the beginning of the treatment is enough to more efficiently inactivate the bacteria. Contrarily, when the antibiotic was not added, the treatment with low MOI was enough to efficiently inactivate the bacteria because, during the first hours of treatment the host efficiently replicates the phages. In fact, the number of phages after 6 h of treatment at MOI of 1 increased more (by 3.4 log PFU/mL in B + P sample) than at MOI of 100 (by 1.8 log PFU/mL). This confirms the hypothesis that, due to the self-perpetuating nature of phages, precise initial doses of phage may not be essential. This is one of the major advantages of phage treatment in relation to antibiotics. On the other hand, a too high MOI can be a disadvantage for the success of phage treatment because the bacteria can be inactivated before replicating the phages, due to the “lysis from without” phenomenon.

In this study, the combination of phages with antibiotics at sublethal and lethal concentrations, do not increase the efficacy of bacterial inactivation relative to the treatment with phages alone. However, the results demonstrated that the combination of antibiotics with phages is an effective alternative to prevent bacterial regrowth when used at 1/5MIC, 1 × MIC or 2 × MIC. The combination with the antibiotic at 1/5MIC delayed the development of resistant bacteria, bacterial regrowth was observed only after 24 h, whereas in the treatment with the phage alone it was observed after 12 h of incubation. When phage ELY-1 was combined with ciprofloxacin at lethal concentrations (1 × MIC and 2 × MIC), after 12 h of treatment, the bacterial reduction by the combined treatment (B + P + Cip1 × MIC and B + P + Cip2 × MIC) was higher than that obtained with the antibiotic alone (B + CipC) but was lower than that caused by the phage alone (B + P). However, when phage was combined with ciprofloxacin at 2 × MIC, the difference was not as high as that observed when the antibiotic was used at MIC. 

The results indicate that the efficiency of the combined therapy with antibiotics and phages depend on the ability of the host bacteria to replicate the phages, which is affected by the antibiotic concentration. In fact, the combined treatment affects the phage production according to the antibiotic concentration. When the ciprofloxacin was added at sublethal concentrations (B + P + Cip1/10MIC and B + P + Cip1/5MIC), the number of phages in the presence of the host increased significantly (2.0 and 0.5–1.0 log PFU/mL, respectively). However, in the treatment with ciprofloxacin at 1 × MIC and 2 × MIC, the concentration of phages remained constant (phage concentration similar to that of phage control). Although the antibiotic addition avoids phage replication by the bacteria when used at high concentrations, the initial quantity of phages was enough to allow the bacterial inactivation. However, the bacterial inactivation in these conditions is delayed relative to the treatment with the phage alone or with the combined treatment with sublethal antibiotic concentrations. 

Considering the results obtained in these experiments, the next step was to evaluate the efficacy of bacterial inactivation using the combined treatment but adding the antibiotic after phage addition (6, 12 and 18 h of phage addition). The *E. coli* was effectively inactivated when the antibiotic was added after the phage for the three tested times but the prevention of bacterial regrowth was more effective when the antibiotic was added after 6 h of phage addition. When the antibiotic was added 12 h after the phage, the bacterial regrowth was prevented, but not as effectively as observed after 6 h and, when the antibiotic was added 18 h after, bacterial regrowth was not prevented. This sequential approach to add the phage and antibiotic in combined treatment allows the bacteria to replicate the phages efficiently. The number of produced phages in the three conditions was similar to that obtained without the antibiotic addition (increase of around 2 log in both conditions) and significantly higher than that observed when the antibiotic was added at the same time as the phage (maximum increase of around 0.5 log). Torres-Barceló et al. (2014) obtained similar results using the combination of streptomycin and the phage LUZ7 against *P. aeruginosa* when the antibiotic streptomycin was added after 12 h of phage addition [36].

The results of this study demonstrated that the combination of antibiotics with phages is an effective alternative to prevent bacterial regrowth, controlling the emergence of resistance to the phages but also to the antibiotic. Similar results have been obtained by other authors [30,36,40,46,49,87] but few of these studies discriminate between prevention of resistance to phages and resistance to antibiotics resulting from the combined treatment [30,36,49]. In this study, we compared not only the resistance of the bacteria to the combined treatment with phages and ciprofloxacin at 1 × MIC and at a sub-inhibitory concentration (1/5MIC), but also the resistance to the phages alone as well as to the antibiotic alone.

The results of this study, as indicated by some other authors [30,42,45], showed that the combined treatment limits the emergence of antibiotic resistant variants. The overall rate of emergence of resistant bacteria was significantly lower in the combined treatment for both ciprofloxacin at 1 × MIC and at 1/5 MIC (4.0 × 10^−7^ and 4.0 × 10^−5^, respectively) than that observed when only the antibiotic was used (3.95 × 10^−6^ and 5.24 × 10^−1^, respectively), for both conditions. Contrarily, other studies demonstrated that phage–antibiotic combinations cause the same resistance as phage and antibiotic introduced individually [36,42,45]. However, to the best of our knowledge, no study indicated that resistance to antibiotics in combined treatment was higher than that developed by the antibiotic alone.

Relative to the emergence of phage-resistant mutants, when ciprofloxacin was added at a MIC together with the phage, the frequency of phage-mutants was lower (4.0 × 10^−7^) than that observed when the antibiotic was not added (3.4 × 10^−5^). However, this reduction was not detected for ciprofloxacin at a sub-inhibitory concentration (1/5MIC). In this case, the concentration of phage-resistant mutants in the presence of ciprofloxacin (4.00 × 10^−5^) was similar to that when phages were tested alone (3.43 × 10^−5^). Nevertheless, in our previous study [30], using another phage of *E. coli*, the addition of ciprofloxacin at a sub-inhibitory concentration (1/10MIC) during phage treatment reduced the emergence of phage-resistant mutants. Further studies, using different antibiotic concentrations and different bacteria and phages, are necessary to clarify the reason for this different behavior.

## 5. Conclusions

We can state that the combined treatment with phages and antibiotics is effective in reducing the bacterial density but also in preventing the emergence of phage-resistant variants. However, the antibiotic concentration and the time of antibiotic application are essential factors to be considered in the combined treatment. In the case of the *E. coli* strain used in this study, when using the combined therapy with the phage ELY-1 and the antibiotic ciprofloxacin, the efficiency of inactivation and the prevention of resistant mutants is higher when the antibiotic is used at 1 × MIC and added 6 h after phage addition. The combined treatment can be used to inactivate *E. coli*, including antibiotic resistant strains, which are among the main ones responsible for moderate and serious infections in hospital and community environments.

## Figures and Tables

**Table 1:** Host range and efficiency of plating of phage ELY-1 determined on 32 bacterial strains. Clear lysis zone (+) and not lysis zone (−).

Species	Infectivity of Phage	Efficacy of Plating (%)
*Escherichia coli* bioluminescent (host)	+	100
*Escherichia coli* AE11	−	0
*Escherichia coli* AN19	−	0
*Escherichia coli* AD6	−	0
*Escherichia coli* AF15	−	0
*Escherichia coli* BC30	+	0
*Escherichia coli* AC5	−	0
*Escherichia coli* AJ23	−	0
*Escherichia coli* BN65	−	0
*Escherichia coli* BM62	−	0
*Escherichia coli* ATCC 25922	+	2.27 × 10^3^
*Escherichia coli* ATCC 13706	−	0
*Enterobacter cloacae*	−	0
*Citrobacter freundii* 6F	−	0
*Proteus mirabilis*	-	0
*Providencia* sp.	-	0
*Salmonella* Typhimurium ATCC 13311	+	3.45 × 10^−3^
*Salmonella* Enteriditis CVA	−	0
*Salmonella* Enteriditis CVB	−	0
*Salmonella* Enteriditis CVC	−	0
*Salmonella* Enteriditis CVD	+	0
*Salmonella* Enteriditis CVE	−	0
*Salmonella* Typhimurium ATCC 14028	−	
*Shigella flexneri* DSM 4782	−	0
*Vibrio parahaemolyticus* DSM 27657	−	0
*Vibrio anguillarum* DSM 21597	−	0
*Aeromonas salmonicida* CECT 894	−	0
*Aeromonas hydrophilla* ATCC 7966	−	0
*Listeria innocua* NCTC 11288	−	0
*Listeria monocytogenes* NCTC 1194	−	0
*Photobacterium damselae damselae* DSM 7482	−	0
*Pseudomonas aeruginosa*	−	0

**Table 2:** Frequency of transformed *E. coli* spontaneous phage-resistant mutants.

Sample	Frequency of Antibiotic-Mutants	Sample	Frequency of Phage and Antibiotic Mutants	Sample	Frequency of Phage-Mutants
Cip 1 × MIC (0.25 µg/mL)	3.95 × 10^−6^	Phage + Cip 1 × MIC	4.04 × 10^−7^	Phage	3.43 × 10^−5^
Cip 1/5MIC (0.05 µg/mL)	5.24 × 10^−1^	Phage + Cip 1/5MIC	4.00 × 10^−5^		
